# Importation of chloroquine-resistant *Plasmodium falciparum* by Guatemalan peacekeepers returning from the Democratic Republic of the Congo

**DOI:** 10.1186/1475-2875-12-344

**Published:** 2013-09-23

**Authors:** Patricia C Juliao, Silvia Sosa, Luis D Gonzalez, Norma Padilla, Lucia Ortiz, Ira Goldman, Venkatachalam Udhayakumar, Kim A Lindblade

**Affiliations:** 1 International Emerging Infections Program, US Centers for Disease Control and Prevention Regional Office for Central America and Panama, 18 Avenida 11-95 zona 15, Vista Hermosa III, 01015 Guatemala, Guatemala; 2 Center for Health Studies, Universidad del Valle de Guatemala, 18 Avenida 11-95 zona 15, Vista Hermosa III, 01015 Guatemala, Guatemala; 3 Military Medical Center of Guatemala, Finca El Palomar, Acatan, Sta. Rosita Zona 16, Guatemala City, Guatemala; 4 Division of Parasitic Diseases and Malaria, Malaria Branch, Center for Global Health, US Centers for Disease Control and Prevention, 1600 Clifton Rd NE, 30030 Atlanta, Georgia, USA

**Keywords:** Malaria, Malaria elimination, Chloroquine resistance, Imported malaria, Guatemala, Military, Central America, Latin America, Democratic Republic of the Congo

## Abstract

**Background:**

Malaria elimination is being pursued in five of seven Central American countries. Military personnel returning from peacekeeping missions in sub-Saharan Africa could import chloroquine-resistant *Plasmodium falciparum*, posing a threat to elimination and to the continued efficacy of first-line chloroquine (CQ) treatment in these countries. This report describes the importation of *P. falciparum* from among 150 Guatemalan army special forces and support staff who spent ten months on a United Nations’ peacekeeping mission in the Democratic Republic of the Congo (DRC) in 2010.

**Methods:**

Investigators reviewed patients’ medical charts and interviewed members of the contingent to identify malaria cases and risk factors for malaria acquisition. Clinical specimens were tested for malaria; isolated parasites were characterized molecularly for CQ resistance.

**Results:**

Investigators identified 12 cases (8%) of laboratory-confirmed *P. falciparum* infection within the contingent; one case was from a soldier infected with a CQ-resistant *pfcrt* genotype resulting in his death. None of the contingent used an insecticide-treated bed net (ITN) or completely adhered to malaria chemoprophylaxis while in the DRC.

**Conclusion:**

This report highlights the need to promote use of malaria prevention measures, in particular ITNs and chemoprophylaxis, among peacekeepers stationed in malaria-endemic areas. Countries attempting to eliminate malaria should consider appropriate methods to screen peacekeepers returning from endemic areas for malaria infections. Cases of malaria in travellers, immigrants and soldiers returning to Central America from countries with CQ-resistant malaria should be assumed to be carry resistant parasites and receive appropriate anti-malarial therapy to prevent severe disease and death.

## Background

Central America has seen a significant decrease in malaria transmission in the last decade; all seven countries (Belize, Guatemala, El Salvador, Honduras, Nicaragua, Costa Rica, and Panama) have experienced >50% decline in the number of confirmed cases between 2000 and 2010 [1]. As a result, five countries (Belize, Nicaragua, El Salvador, Panama, and Costa Rica) are designated as malaria eliminating [2], although all countries have joined a regional initiative that aims to eliminate malaria by 2020 [3].

As the incidence of malaria declines in Central America to near zero, imported malaria cases pose a threat to the achievement of elimination. Surveillance systems will need to rapidly detect, classify and treat imported cases before onward transmission occurs. The potential for introduction of chloroquine (CQ)-resistant *Plasmodium falciparum* from other parts of the globe could further threaten elimination as most countries in Central America still rely on CQ, along with primaquine, as first-line treatment for both *P. falciparum* and *Plasmodium vivax*[4].

Although civilian travellers and migrants are the populations most often considered as sources of imported malaria cases to non-endemic countries, military personnel returning from deployment to malaria-endemic areas also contribute to malaria importation [5]. Central American militaries participate in United Nations (UN) missions in politically troubled areas of Africa. Currently there are seven UN peacekeeping missions in Africa, and six of these are in malaria-endemic countries; Central American soldiers participate in all of these missions [6]. Due to the large number of soldiers and frequency of deployment, UN peacekeepers could serve as a significant source of malaria imported into Central America.

Malaria transmission occurs in rural areas of Guatemala at altitudes below 1,500 m, excluding the highlands around Lake Atitlán; approximately 70% of the total area of the country is considered endemic; Guatemala City is not within the malaria-endemic area [7]. However, transmission of malaria in Guatemala is low; the Guatemala Ministry of Public Health and Social Welfare (MOPH) estimates annual malaria incidence (both *P. falciparum* and *P. vivax*) in endemic areas of the country to be 2.2 cases per 1,000 population per year [8]. In 2010, 7,314 cases of *P. vivax* and 31 of *P. falciparum* were reported nationally*.* Although no therapeutic efficacy studies have been conducted, all samples of *P. falciparum* from cases in Guatemala in 1998 and 2001 that were tested for CQ resistance genes presented with the wild type, confirming CQ susceptibility (pers. comm., N Padilla).

In October 2010, two soldiers from the Guatemalan *Kaibiles* (special forces) recently returned from a UN peacekeeping assignment in the Democratic Republic of the Congo (DRC) were hospitalized with severe malaria, and one of them died from his infection. This report describes the active investigation of malaria among the peacekeeping contingent, including molecular characterization of *P. falciparum* genotypes associated with CQ resistance.

## Methods

### Study population

A contingent of 144 Guatemalan soldiers and 6 civilian support staff deployed to the DRC in January 2010 and returned to Guatemala City on 17 October, 2010. The contingent was comprised of males between the ages of 19 and 55 and their home base in Guatemala was at Mariscal Zabala, located in Guatemala City. After returning to Guatemala City, the contingent was granted leave on 22 October, 2010 and all members returned to their homes throughout Guatemala.

### Case definition

Investigators defined an imported case of malaria as either microscopy-confirmed *P. falciparum* in a thick or thin blood smear or a dried filter-paper blood sample positive for *P*. *falciparum* using nested polymerase chain reaction (PCR), occurring in a member of the DRC contingent within three weeks of their return to Guatemala (between 17 October and 12 November, 2010). Clinical malaria was defined as measured fever (axillary temperature ≥38°C) or history of fever from two weeks before leaving the DRC (1 October, 2010) to date of interview (5 to 12 November, 2012) in a member of the contingent with confirmed *P. falciparum* infection.

### Case identification

The first two cases were identified when they presented with signs of severe malaria and were hospitalized at the Military Medical Center in Guatemala City. Military medical personnel requested technical assistance from the US Centers for Disease Control and Prevention (CDC) and the Center for Health Studies, Universidad del Valle de Guatemala (CES-UVG) in the management and control of this malaria importation event. Active case detection was undertaken by CDC and CES-UVG among the remaining members of the contingent after they had been recalled to Guatemala City on 5 November, 2012.

Every member of the contingent was interviewed to determine his history of clinical symptoms from two weeks before leaving the DRC to the date of interview (a period ranging between five and six weeks) along with use of malaria prophylaxis and mosquito avoidance measures, including insecticide-treated bed nets (ITNs), travel history within the DRC and travel upon return to Guatemala. All members of the contingent had been provided malaria prophylaxis with mefloquine (MQ) prior to departure. Correct adherence to MQ prophylaxis was defined as one dose of MQ every week beginning two weeks before arrival in the DRC and ending four weeks after return to Guatemala [9]. Malaria-endemic areas within Guatemala were defined by the Ministry of Health as an area with documented malaria cases and documented presence of known malaria vectors.

### Laboratory testing

Blood smears were stained with Giemsa and examined for parasites by microscopists at the CES-UVG. During the active screening, finger-prick blood samples for thick and thin blood smears and dried blood spots on filter paper were obtained from all participants; rapid diagnostic tests (OptiMAL –IT, BioRad Laboratories, Switzerland) were used for anyone with a history of fever in the previous 48 hours to allow immediate treatment if necessary. Parasite density was calculated by counting parasites against 1,000 × white blood cells (WBCs) and assuming 8,000 WBCs per μl of blood. A slide was determined to be negative after reviewing 100 fields without finding parasites. Patients found positive for malaria during the active investigation were treated with artemisinin-based combination therapy according to international guidelines [10].

All dried blood spots, whole blood, and *post-mortem* tissue specimens from the case-patient who died were screened with PCR for *P. falciparum.* Extraction of DNA from these samples was accomplished using the QIAamp DNA Minikit (Qiagen, Inc, Valencia, CA, USA) following the manufacturer’s instructions. A nested PCR targeting the 18S small subunit ribosomal RNA gene of *P. falciparum* was used [11,12]; positive results were retested once in Guatemala and then reconfirmed by the CDC laboratory in Atlanta, GA, USA. The DNA from the *P. falciparum*-positive samples was sequenced to determine the presence of CQ-resistant genotypes, specifically, polymorphisms in codons 72–76 of the *pfcrt* gene. A semi-nested PCR was used to amplify the *pfcrt* gene in each sample [13-15]. Outer PCR reaction was diluted 1:100 for positive cases. PCR products were purified using ExoSap reaction [16] and were sent for sequencing (Macrogen Inc, USA and Macrogen Inc, Seoul, Korea). Sequences were aligned with reference strains using MEGA5 software [17] to identify the polymorphisms.

### Human subjects

The investigation was initiated after the hospitalization of the first two cases to prevent additional cases of severe disease as well as onward transmission of malaria within Guatemala; the investigation received human subjects review and was determined to be a public health response. Verbal consent was obtained for blood sampling and testing as well as interviews to determine illness history and use of malaria prevention measures.

## Results

Two cases of severe febrile illness among the DRC contingent members were identified by physicians at the Military Medical Center. One of the soldiers died after being treated for two days with CQ and primaquine. Parasitologic confirmation of *P. falciparum* as the cause of the second soldier’s febrile illness was obtained within six days of the death of the first soldier and prompted the return of the remaining contingent to base camp.

Thick and thin blood slides, whole blood and filter-paper blood spots were obtained from 149 (99%) members of the contingent, along with three *post-mortem* tissue samples from the deceased case-patient. Twelve cases (8%) had laboratory confirmation of *P. falciparum* infection, including the deceased case-patient (Table 1). Five of the 12 cases (42%) were positive by both microscopy and PCR, and seven of 12 (58%) were positive by PCR alone (Table 1). Four cases had thick smears that could be used to calculate parasite density, which ranged from 2,152 to 71,408 parasites per μl; none of the cases positive by microscopy had detectable gametocytes. All of the five cases that had patent parasitaemia, but none of the seven subpatent cases that were PCR-positive only, reported a history of fever. Symptom onset dates ranged from 12 October, 2010 (five days before leaving DRC) to 7 November, 2010 (three weeks after arrival in Guatemala).

**Table 1:** Results of laboratory testing for 12 malaria-infected members of the Guatemalan peacekeeping contingent

**Px**	**Age, year**	**Symptom onset**	**Sample collected**	**Laboratory confirmed**^**d**^	**Thin/thick blood smears**			**PCR result**	***Pfcrt *haplotype**^**f**^
					**Parasitaemia**	**Parasite density**^**e**^	**Gametocytes**		
1^a^	37	10/23/2010	10/27/2010	11/08/2010	(+)	UA	UA	(+)	CV**IET**^**g**^
2	28	10/30/2010	11/03/2010	11/04/2010	(+)	71,408	(−)	(+)	CVMNK
3	22	10/13/2010	11/09/2010	11/09/2010	(+)	6,008	(−)	(+)	CVMNK
4^b,c^	44	11/07/2010	11/09/2010	11/10/2010	(+)	18,200	(−)	(+)	CVMNK
5	22	10/12/2010	11/09/2010	11/12/2010	(+)	2,152	(−)	(+)	CVMNK
6	48	AI	11/09/2010	11/29/2010	(−)	na	na	(+)	CVMNK
7	25	AI	11/09/2010	11/29/2010	(−)	na	na	(+)	CVMNK
8	30	AI	11/09/2010	11/29/2010	(−)	na	na	(+)	CVMNK
9	22	AI	11/09/2010	11/29/2010	(−)	na	na	(+)	CVMNK
10	28	AI	11/12/2010	11/29/2010	(−)	na	na	(+)	CVMNK
11	26	AI	11/09/2010	11/29/2010	(−)	na	na	(+)	CVMNK
12	25	AI	11/09/2010	11/29/2010	(−)	na	na	(+)	CVMNK

Px: patient; na: not applicable; AI: asymptomatic infection; UA: data unavailable.

^a^Parasite density cannot be calculated; PCR results were obtained from *post-mortem* tissue.

^b^Patient was initially diagnosed with *P. falciparum* by the OptiMAL-IT Rapid test.

^c^Patient reported a previous malaria infection in the DRC; received artequine as treatment.

^d^Dates corresponds to thin/thick blood smears results for patient 1–5 and PCR results for patients 6-12.

^e^No of asexual parasites/μl of blood.

^f^CQ resistance type in the *pfcrt* gene: CVMNK (wild type); CV**IET** (CQ resistant).

^g^Sequencing results showed a major peak for CVIET and minor peak for CVMNK when retested.

All 12 of the malaria-positive samples were tested for the presence of CQ-resistant *P. falciparum pfcrt* genotype (Table 1). One (8%) out of the 12 samples had a triple mutant haplotype (CVIET) previously reported in DRC [18]; this sample was obtained from the deceased soldier. It is worth noting that this patient sample also contained a minor population of parasite with CQ-sensitive CVMNK, although CQ-resistant CVIET allele was predominant. The remaining 11 (92%) samples had the wild type CQ-sensitive *pfcrt* genotype CVMNK.

Among the 149 individuals interviewed, 147 (99%) reported using a bed net while stationed in the DRC (Table 2). However, none reported using a bed net treated with an insecticide. A total of 141 (82%) persons reported using a bed net every night during the last two weeks in the DRC and 142 (82%) reported using mosquito repellent at least once a day during their stay in the DRC. Although all 149 individuals reported taking MQ prophylaxis, none had adhered completely to the regimen (Table 2). A total of 17 (11%) persons reported being diagnosed with and treated for malaria during their time in the DRC; one (6%) of those reporting a previous malaria diagnosis was malaria positive at the time of the investigation.

**Table 2:** Reported risk factors associated with malaria among the contingent of Guatemalan peacekeepers, 2010

**Characteristics**	**Imported cases,**		**Non-cases,**	
	**N =11**^**a**^		**N=138**	
	**n**	**(%)**	**n**	**%**
**Use of bed nets**				
Used a bed net during stay in the DRC	11	(100)	136	(94)
Used a bed net every night for the last two weeks in DRC	10	(91)	131	(90)
Used an insecticide-treated bed net	0	(0)	0	(0)
**Use of mosquito repellent**				
Used mosquito repellent during stay in the DRC	10	(91)	132	(91)
Used at least once a day	8	(73)	114	(83)
**Adherence to malaria chemoprophylaxis**				
Adhered to MQ prophylaxis while in the DRC^b^	8	(73)	64	(44)
Adhered to MQ prophylaxis overall^c^	0	(0)	0	(0)

^a^Data unavailable for patient 1.

^b^MQ = mefloquine. Adherence to chemoprophylaxis in DRC defined as one dose of MQ every week for at least eight months while in the DRC.

^c^Overall adherence defined as: one dose of MQ every week beginning two weeks before arrival in the DRC and ending four weeks after return to Guatemala.

Of the 12 patients with confirmed malaria, eight (67%) reported visiting at least one area endemic for malaria within Guatemala after their return from the DRC. Three of the eight had clinical malaria but the dates of their symptom onset did not suggest they were infected in Guatemala. The five remaining persons had subpatent infections with no symptoms and therefore it is feasible that the infection may have been acquired in Guatemala. However, due to the low *P. falciparum* malaria endemicity levels in Guatemala it is likely the infection was acquired in the DRC [8].

## Discussion

Investigators identified 12 cases of *P. falciparum* imported to Guatemala from one contingent of soldiers and civilians supporting a UN peacekeeping mission in the DRC. More than half of the malaria cases had no history of fever in the preceding five to six weeks, and only one reported a malaria diagnosis while deployed in the DRC. One patient, who subsequently died, was infected with a typical CQ-resistant genotype of *P. falciparum* along with a minor population of CQ-sensitive CVMNK allele probably acquired in DRC, which was not recognized in time to provide appropriate, potentially life-saving treatment. This finding also suggests that this patient may have acquired *P. falciparum* infection more than once in the past as both CQ-resistant and a minor population of CQ-sensitive alleles were detected in this patient. Alternatively, this patient may have been bitten by a mosquito that carried both CQ-resistant and CQ-sensitive parasites. The presence of CVIET genotype in the DRC has been documented previously [19] and this is consistent with the epidemiologic data that confirm that the soldiers and support staff acquired these *P. falciparum* infections in the DRC. A future publication will compare microsatellite markers on the imported parasites with endemic strains from both Guatemala and the DRC to further support the DRC as the source of the infections.

No member of the Guatemalan contingent had completely adhered to malaria chemoprophylaxis recommendations or used an ITN during deployment in the DRC. Lack of adherence to chemoprophylaxis is a risk factor for malaria infection when travelling in malaria-endemic areas [20]. ITNs have been proven to reduce risk of infection from malaria in multiple trials [21] and are recommended for use by travellers to malaria-endemic areas [9]. Malaria prophylaxis for peacekeepers and support staff should be directly observed, and soldiers and support staff provided with ITNs and insect repellent for personal protection. Most importantly, countries where CQ remains the first-line therapy should be prepared for CQ-resistant infections in travellers, immigrants and soldiers who return from areas where CQ is no longer effective. All malaria infections acquired in areas with CQ resistance should be treated with an appropriate anti-malarial to prevent severe disease and onward transmission.

Similar preventive and control measures have been implemented elsewhere in response to imported malaria events associated with peacekeeper or humanitarian missions. In 2000, the US military recommended directly observed therapy for malaria chemoprophylaxis for military personnel stationed in highly malaria endemic areas [22,23]. Other preventive practices that have been implemented to reduce the number and impact of malaria infections occurring during military deployments include: equipping troops with permethrin-impregnated uniforms and insect repellent containing DEET; screening military personnel upon return to their home country; training health care providers within the home country on malaria diagnostics and treatment; and administration of a prophylactic course of primaquine at the end of deployment [22-25]. All measures include increases in training of military personnel on the risk of malaria, and proper use of personal protective gear and chemoprophylaxis before, during, and after deployment.

Despite widespread CQ resistance in *P. falciparum* in Africa, Asia and South America, *P. falciparum* parasites circulating in Central America north of the Panama Canal retain susceptibility to CQ [26,27]. It is not clear why CQ-resistant parasites, found from just south of the Panama Canal to Brazil, have not yet penetrated Central America. It has been suggested that the susceptibility of *Anopheles* mosquitoes may differ by parasite strain, and by extension, local vector populations in Central America may be refractory to foreign *Plasmodium* strains, limiting their onward transmission [28,29]. However, this hypothesis has not been proven, and there are examples of drug resistance spreading from importations of resistant parasites [30,31]. Increasing global tourism and frequent travel of unprotected Central American residents to malaria-endemic regions may increase the risk of importation of CQ-resistant parasites into Central America. Military peacekeeping missions that do not ensure adherence to malaria prevention measures are at significant risk of importing resistant parasites from Africa to Central America due to the frequency of their deployment, the relatively large numbers of individuals deployed, and the length of time spent in malaria-endemic areas.

Only eight members of the affected military contingent travelled to malaria-endemic areas of Guatemala while infected. October, the month of the soldiers’ return, is at the end of the rainy season when temperatures begin to cool, which would limit both the numbers of malaria vectors present and the development of *P. falciparum,* were any anopheline mosquitoes to have been infected. Therefore, the risk of onward *P. falciparum* transmission from this contingent of peacekeepers was likely to have been low. However, these 12 imported cases comprise almost 30% of the total number of *P. falciparum* cases diagnosed in 2010 in Guatemala. While malaria cases are under-reported through the health information system to the Guatemala MOPH, imported *P. falciparum* cases are likely to make up a significant fraction of the *P. falciparum* cases treated in Guatemala any given year.

The potential for recall bias to have affected report of malaria prevention measures by members of the contingent with and without malaria was investigated, but considered unlikely as most members of the contingent were interviewed before their malaria status was known, and use of prophylaxis and personal prevention measures was uniformly low. This paper reports on the experiences of one contingent of peacekeeping forces and support staff to the DRC from Guatemala, and is not necessarily representative of all contingents from all countries. However, the recommendations in this paper will be applicable to all travellers, visitors and military contingents travelling in malaria-endemic areas of sub-Saharan Africa.

As countries in Central America begin to work towards malaria elimination, importation of malaria parasites, especially those resistant to first-line therapy, poses several challenges to eventual elimination. Population movements have resulted in setbacks in malaria control in many countries [30,32]. To avoid the potential impact of malaria importation on elimination, countries should strengthen surveillance systems to be able to rapidly identify imported cases to prevent onward transmission. Given the potential for military peacekeeping missions deployed in malaria-endemic regions to return with infections resistant to first-line therapy in their home country, thereby posing a threat to their own health as well as the potential for local transmission of resistant parasites, it is recommended that Central American countries consider evaluating strategies to screen returning peacekeeping missions for malaria.

## Competing interests

The authors declare that they have no competing interests.

## Authors’ contributions

PJ coordinated the epidemiologic investigation of the importation event. PJ, SS, LD, and KL participated in case identification, survey design and administration. NP and LO carried out malaria diagnostics on blood and tissue specimens and NP, LO, VU, and IG conducted CQ resistance genotyping of the malaria parasite. Both PJ and KL drafted the manuscript. All authors read and approved the final manuscript.
